# Mutations for Leber hereditary optic neuropathy in patients with alcohol and tobacco optic neuropathy

**Published:** 2011-12-07

**Authors:** Marcela Scabello Amaral-Fernandes, Ana Maria Marcondes, Paulo Maurício do Amor Divino Miranda, Andréa Trevas Maciel-Guerra, Edi Lúcia Sartorato

**Affiliations:** 1 Departamento de Oftalmologia e Otorrinolaringologia, Faculdade de Ciências Médicas, Universidade Estadual de Campinas, São Paulo, Brasil; 2 Centro de Biologia Molecular e Engenharia Genética, Universidade Estadual de Campinas, São Paulo, Brasil; 3 Departamento de Genética Médica, Faculdade de Ciências Médicas, Universidade Estadual de Campinas, São Paulo, Brasil

## Abstract

**Purpose:**

There are many similarities in the clinical presentation of Leber hereditary optic neuropathy (LHON) and in patients who have optic neuropathy and a history of heavy tobacco and alcohol consumption. The main objective of this study is to investigate the frequency of primary and secondary mitochondrial DNA (mtDNA) mutations for LHON in patients diagnosed as having alcohol and tobacco optic neuropathy (ATON).

**Methods:**

Twenty-six patients who had a history of heavy alcohol and tobacco consumption and who developed bilateral optic neuropathy were tested for primary mutations (G11778A, T14484C, and G3460A) by restriction analysis, and 14 secondary mutations in the genes mitochondrially encoded NADH dehydrogenase 1 (*MT-ND1*), mitochondrially encoded NADH dehydrogenase 4 (*MT-ND4*), mitochondrially encoded NADH dehydrogenase 4L (*MT-ND4L*), mitochondrially encoded NADH dehydrogenase 5 (*MT-ND5*), mitochondrially encoded NADH dehydrogenase 6 (*MT-ND6*), and mitochondrially encoded cytochrome B (*MT-CYB*) by direct sequencing.

**Results:**

Four (15.4%) of 26 patients tested positive for LHON primary mutations, two for the G11778A mutation, and two for the T14484C mutation. No patient tested positive for any of the 14 secondary mutations. Familial recurrence was present in four patients, and only three of these patients have presented the LHON mutation.

**Conclusions:**

The diagnosis of LHON should be considered in all patients diagnosed as having optic neuropathy, particularly those with familial recurrence of vision loss.

## Introduction

Leber hereditary optic neuropathy (LHON) is a maternally inherited mitochondrial disease characterized by a bilateral acute or subacute painless loss of vision, central or cecocentral scotoma, and dyschromatopsia [1].

Most LHON patients have a profound decrease of visual acuity of less than 20/200 to counting fingers range and visual loss occurs simultaneous in both eyes or in one eye initially, which is followed by visual loss in the other eye within weeks to months [2,3]. LHON usually affects healthy young male adults in the second and third decades of life, with a mean age at onset of 22 years [4], ranging from 5 to 81 years [5,6]. Seventeen mutations related to LHON have so far been studied, G11778A, G3460A, and T14484C account for 95% of cases (primary mutations); the other 14 mutations are secondary mutations (T4160C, C4171A, G11696A, T11253C, T10663C, A13637G, G13730A, G14459A, C14482G, A14495G, T14898C, C14568T, G14596A, G15257A) [7-9]. Three mitochondrial DNA (mtDNA) point mutations, at positions 11778/ND4, 3460/ND1, and 144484/ND6, are primary mutations responsible for the large majority of cases reported [2,4]. These three point mutations are located in the genes of nicotinamide adenine dinucleotide hydride (NADH) dehydrogenase subunits of complex 1 of the respiratory chain. These mutations may either decrease the ATP synthesis for a cell or induce a chronic overproduction of reactive oxygen species in the mitochondria. The resultant oxidative stress may trigger apoptosis in the cell [3]. The degeneration of the optic nerve is due to a loss of retinal ganglion cells and optic nerve axons, with a preferential loss of p-ganglion cells, which are responsible for central vision [4]. LHON has an incomplete penetrance, and chances for carriers of a LHON mutation to become affected are approximately 30% to 50% for male subjects and 10% to 15% for female subjects. The mutation in the mtDNA is essential but it alone is not enough to initiate the disease. Secondary genetic and/or epigenetic or environmental risk factors might play a role in the pathogenesis. Nonhereditary causes, such as smoking, alcohol, drug use, trauma, or vascular causes, have been postulated as risk factors for the phenotypic expression of LHON [4,10].

The optic neuropathy in patients with heavy tobacco–alcohol consumption is characterized by subacute painless loss of visual acuity and centrocecal scotomas occurring most frequently in men with heavy tobacco use, alcohol use, or both [11,12]. There are many similarities in the clinical presentation of LHON and in patients who have optic neuropathy and a history of heavy tobacco and alcohol consumption. The pathogenesis of the disease is supposed to be secondary to a nutritional deficiency or the direct toxic effect of alcohol, tobacco, or both. The cause of alcohol and tobacco optic neuropathy (ATON) has not yet been proven; however an underlying susceptibility that predisposes patients to this condition has been postulated [12]. Since there are many similarities in the clinical presentation of LHON and ATON, the investigation of molecular genetic LHON mtDNA mutations is essential for the differential diagnosis between these two neuropathies [12].

## Methods

### Patients

Between August 2007 to November 2009, 26 patients from the Departamento de Oftalmologia da Faculdade de Ciências Médicas - Universidade Estadual de Campinas (UNICAMP, Campinas, São Paulo, Brazil) were examined. Informed consent was obtained from participants before the study, conforming to the tenets of the Declaration of Helsinki and following the Guidance for Sample Collection of Human Genetic Disease (National 863-Plan) by the Research Ethics Committee/Faculdade de Ciências Médicas - Universidade Estadual de Campinas, UNICAMP (number: 690/2004). The inclusion criteria were: history of heavy tobacco and alcohol consumption (100 ml of distilled alcohol per day during 1 year or more) in patients older than 18 years, (sub) acute visual loss (less than 20/40), and central or cecocentral scotomas. Patients with ocular surgery or any other pathology that could cause visual loss were excluded as were those with metabolic disease (diabetes mellitus). Computerized tomography or magnetic resonance of the brain was used to detect any other neurologic condition.

### Methods

Complete eye examinations were performed, and peripheral blood samples (5 ml, blood drawn by venipuncture) were collected in EDTA vacutainers (Greiner Bio-one Catalogue no. 455036; Frickenhausen, Germany). The sample was sent at 4 °C to the laboratory of Centro de Biologia Molecular e Engenharia Genética (CBMEG) - UNICAMP for mtDNA analysis.

Genomic DNA was extracted from venous blood samples using standard phenol–chloroform extraction. Molecular genetic analysis was performed using PCR amplification of DNA extracted from peripheral blood leucocytes [13]. The presence of the G3460A, G11778A, and T14484C mutations was examined by PCR-Polymerase Chain Reaction -Restriction fragment length polymorphism (RFLP) with restriction endonuclease digestions. The secondary mutations in genes mitochondrially encoded NADH dehydrogenase 1 (*MT-ND1*), mitochondrially encoded NADH dehydrogenase 4 (*MT-ND4*), mitochondrially encoded NADH dehydrogenase 4L (*MT-ND4L*), mitochondrially encoded NADH dehydrogenase 5 (*MT-ND5*), mitochondrially encoded NADH dehydrogenase 6 (*MT-ND6*), and mitochondrially encoded cytochrome B (*MT-CYB*) *w*ere screened by direct sequencing using an ABI prism Big Dye Terminator cycle sequencing Ready Reaction kit V 3.1 (ABI Prism/ Apllied Biosystems, Foster City, CA) and analyzed on an ABI Prism 3100 Genetic Analyzer (Applied Biosystem). All sequences were analyzed against mitochondrial reference sequence NC_012920.

## Results

Twenty-five (96.2%) male patients and one female patient (3.8%) were tested (Table 1). The mean age at onset was 38 years, ranging from 17 to 52 years. All 26 (100%) patients had a history of heavy alcohol consumption and 25 (96.2%) had a history of tobacco abuse, at least 1 year before the patients had manifested the loss of vision and optic neuropathy. Visual acuity ranged from 20/50 to counting finger at 1 m. All patients showed scotomas in the visual field in static perimetry, two patients (7.7%) showed relative central scotoma, and 24 patients (92.3%) showed central or ceccocentral scotomas. When Ishihara’s test was performed, all patients showed abnormal results. Four (15.4%) of 26 patients tested positive for LHON primary mutations, two for the G11778A mutation, and two for the T14484C mutation. Three of the four mutant homoplasmic patients (4, 8, and 15) had a maternal antecedent of visual loss. These patients were not related. None of the 26 patients tested positive for any of the 14 secondary mutations analyzed.

**Table 1:** Summary of clinical and molecular data of the patients studied.

**Patient/gender**	**Age of manifestation**	**Visual acuity**	**Visual fields**	**Familial recurrence**	**Mutation**
1/M	35	OD 20/200	Cecocentral	-	-
		OS 20/200	Cecocentral		
2/M	35	OD 20/100	Central	-	-
		OS 20/50	Central		
3/M	35	OD 20/100	Central	-	-
		OS cf at 1m	Central		
4/M	47	OD 20/200	Cecocentral	+	T14484C
		OS 20/200	Cecocentral		
5/M	46	OD 20/50	Relative central scotoma	-	-
		OS 20/60	Relative central scotoma		
6/M	42	OD 20/200	Cecocentral	-	-
		OS 20/200	Cecocentral		
7/M	52	OD 20/200	Central	-	-
		OS 20/100	Central		
8/M	35	OD cf at 1m	Fovea 4dB	+	G11778A
		OS cf at 1m	Fovea 0dB		
9/M	46	OD 20/100	Central	-	-
		OS 20/100	Central		
10/M	38	OD 20/100	Central	-	-
		OS 20/100	Central		
11/M	43	OD cf at 1m	Central	-	-
		OS cf at 1m	Central		
12/M	44	OD 20/50	Relative central scotoma	-	-
		OS 20/50	Relative central scotoma		
13/M	41	OD 20/200	Fovea 9dB	-	-
		OS 20/200	Fovea 13dB		
14/M	52	OD cf at 1m	Cecocentral	-	-
		OS cf at 1m	Cecocentral		
15/M	27	OD cf at 1m	Central	+	T14484C
		OS cf at 1m	Central		
16/M	40	OD 20/50	Central	-	-
		OS 20/50	Central		
17/M	50	OD cf at 1m	Fovea 5dB	-	-
		OS 20/200	Fovea 0dB		
18/M	44	OD cf at 1m	Fovea 10dB	-	-
		OS cf at 1m	Fovea 10dB		
19/M	17	OD 20/100	Central	+	-
		OS 20/200	Central		
20/M	41	OD 20/200	Central	-	-
		OS 20/50	Central		
21/M	29	OD 20/50	Central	-	-
		OS 20/50	Central		
22/M	40	OD 20/60	Central	-	-
		OS 20/100	Cecocentral		
23/M	33	OD 20/100	Central	-	-
		OS 20/100	Central		
24/M	38	OD 20/60	Central	-	-
		OS 20/60	Central		
25/M	17	OD cf at 1m	Fovea 0dB	-	G11778A
		OS cf at 1m	Cecocentral		
26/F	20	OD 20/50	Central	-	-
		OS 20/100	Cecocentral		

Abbreviations: OD represents oculus dexter, right eye; OS represents oculus sinister, left eye; Cf represents counting finger (it is a range, a measurment of visual acuity); dB represents decibel.

## Discussion

It is known that several primary inherited disorders of mitochondrial dysfunction target the optic nerve, including LHON. In addition, disorders of secondary mitochondrial dysfunction also target the optic nerve. These include dominant optic atrophy, ATON, and chloramphenicol optic neuropathy [14,15].

Mitochondrial energy production decreases with age, and the timing of visual loss in patients at risk for LHON may reflect the threshold at which the already reduced mitochondrial function deteriorates to a critical level [16].

The possibility that environmental factors, both internal and external, could trigger vision loss in susceptible patients with LHON has been based on the hypothesis that vision loss is linked to defects in oxidative phosphorylation [14]. Systemic illnesses, immunologic factors, nutritional deficiencies, medications, or toxins that stress or directly inhibit mitochondrial metabolism could conceivably initiate or increase phenotypic manifestation of the disorder. Despite the great advances in our understanding of mitochondrial genetics and disease over the past decade, the underlying pathophysiological mechanisms linking the specific gene defect with the clinical manifestation of LHON remain elusive [16].

ATON and LHON have similar clinical characteristics and the pathogenesis of the visual loss in the two diseases may be related. Patients who have LHON may be misdiagnosed as having ATON when in fact environmental insults are allowing the clinical expression of a disease that otherwise would go unnoticed.

It has been noted that although a substantial percentage of the population uses alcohol, relatively few persons develop visual loss. Conversely, some patients who consume relatively small amounts of alcohol develop a bilateral optic neuropathy thought to be caused by the toxic effects of alcohol [12]. Many authors have studied the correlation between abusive use of tobacco and alcohol and the visual loss in mutant patients for LHON [12,17]. A recent multicenter study identified an association between smoking and visual loss, and this relationship might even be dose responsive. The authors also identified an increased visual failure with alcohol consumption but only with heavy intake of alcohol [18].

In our study, 26 patients who had a history of heavy alcohol consumption and developed bilateral optic neuropathy were tested for the three primary (G117778A, T14484C, and G3460A) and 14 secondary LHON mtDNA mutations. The genetic molecular analysis tested positive for LHON in four patients, two patients tested positive for the G11778A mutation, and two patients tested positive for T14484C mutations. Three of these patients had a family history of vision loss in maternal relatives, and the other patient had no information about his relatives. The absence of secondary mutations in our study is concordant with the literature because these mutations are responsible for less than 5% of the cases of LHON [7,9]. Our first hypothesis was not LHON for these four mutant patients because they had atypical clinical presentation. Three of the patients had subacute visual loss during a period of 6 months to 2 years. The fourth patient lost his vision at 17 years but started to drink and smoke when he was 14 years old.

Despite all patients having had heavy alcohol consumption during at least 1 year and optic neuropathy, the mutations for LHON were detected only in patients with a maternal antecedent of visual loss.

In this study we cannot make any definitive statement between alcohol and LHON manifestation. Otherwise, only patients with a family history showed the mutation for LHON.

We could suggest that epigenetic factors, more so than environmental risk factors, would be determinants in the phenotypic expression of LHON, such as the co-existence of homoplasmic mutation, the presence of nuclear-encoded factors, and an X-linked modifier locus [16]. The underlying mtDNA haplotype may also influence the presence, penetrance, or expression of a mtDNA point mutation and disease severity [4,16].

New studies will be necessary to better understand the importance of genetic, epigenetic, and environmental factors in LHON expression. There are no studies at the moment clearly confirming the influence of smoking and heavy alcohol consumption in the expression of visual loss in mitochondrial optic neuropathy. Alcohol and tobacco may trigger visual loss by other mechanisms besides mtDNA mutation for LHON, and this is possibly related to epigenetic factors.

The present study highlights the importance of careful anamnesis focusing on familial recurrence of visual loss in patients with optic neuropathy to indicate molecular genetic analysis. Genetic counseling of families with LHON mutations and advice on smoking avoidance and alcohol intake are important to prevent visual loss.
